# Improving the quality of care of respiratory syncytial virus in the neonatal and pediatric populations globally

**DOI:** 10.3389/fped.2025.1590842

**Published:** 2025-05-21

**Authors:** Fungwe Jah, Asuncion Mejias, Tendai Nzirawa, Rita C. Silveira, Angelika Berger, Laura Travan, Daisuke Kinoshita, Shareena Ishak, Tolga Çelik, Faisal Al-dandan, Benedikt Mahr, Christine Masters

**Affiliations:** ^1^ Medical Affairs, BioPharmaceuticals Medical, AstraZeneca, Hamburg, Germany; ^2^ Department of Infectious Diseases, St Jude Children’s Research Hospital, Memphis, TN, United States; ^3^ Paediatric Pan London Oxygen Group (PPLOG), London, United Kingdom; ^4^ Department of Pediatrics - Newborn Section, Federal University of Rio Grande do Sul and Hospital de Clinicas de Porto Alegre, Porto Alegre, Rio Grande do Sul, Brazil; ^5^ Center for Pediatrics, Department of Pediatrics and Adolescent Medicine, Medical University of Vienna, Vienna, Austria; ^6^ Department of Neonatology, Institute for Maternal and Child Health IRCCS Burlo Garofolo, Trieste, Italy; ^7^ Department of Neonatology, Japanese Red Cross Kyoto Daiichi Hospital, Kyoto, Japan; ^8^ Department of Paediatrics, Faculty of Medicine, National University of Malaysia, Kuala Lumpur, Malaysia; ^9^ Division of Neonatology, Department of Pediatrics, Hacettepe University Faculty of Medicine, Ankara, Türkiye; ^10^ Department of Neonatology, Maternity and Children Hospital Al-Ahsa, Al-Mubarraz, Al-Ahsa, Saudi Arabia; ^11^ Medical Affairs, BioPharmaceuticals Medical, AstraZeneca, Vienna, Austria; ^12^ Medical Affairs, BioPharmaceuticals Medical, AstraZeneca, London, United Kingdom

**Keywords:** pediatrics, neonatology, infants, respiratory infections, respiratory syncytial virus

## Abstract

Respiratory syncytial virus (RSV) is the leading cause of hospitalization for bronchiolitis in infants worldwide, and age (<6 months) and underlying comorbidities (e.g., prematurity, congenital heart disease) are risk factors for severe disease. However, some centers face challenges in identifying and implementing preventative measures, and best practices, for the care of at-risk infants. Therefore, this study aimed to identify best practice examples in RSV care for neonatal and pediatric populations in leading centers globally, and to understand how these practices can be widely implemented. Following a literature review, multidisciplinary teams were interviewed in 10 centers globally (1 center per country; 40 interviews conducted between May and November 2023). Centers were included based on pre-determined criteria (e.g., type of center, services provided, focus on RSV research) to ensure a representative view of RSV care. The identified best practice interventions were critically reviewed by a group of RSV experts [healthcare professionals (HCPs) and a patient group representative] and assessed for their impact on patient care and transferability to other centers. Fifty-seven unique best practice interventions were identified, sixteen of which were prioritized, across five best practice themes: (1). Caregiver education and engagement: Provision of timely caregiver education on RSV infection and care. (2). HCP education: Provision of continuous evidence-based HCP education. (3). HCP-led RSV prophylaxis services: Additional support services to ensure at-risk infants are protected ahead of the RSV season. (4). Protocols and ways of working: Establishing evidence-based procedures to ensure best practices are followed within clinical practice. (5). Technology and innovation: Leveraging digital services to optimize care delivery and experience. This study identified interventions that may improve patient outcomes and quality of care for RSV disease in the pediatric and neonatal populations. The next steps will be to disseminate and implement best practice examples across healthcare systems and care settings globally.

## 1 Introduction

Respiratory syncytial virus (RSV) is the main cause of hospitalization for lower respiratory tract infections in infants and young children worldwide and is a leading cause of mortality and morbidity in children < 5 years (1–3). Notably, RSV is also the leading cause of pneumonia in children < 5 years (4).

Risk factors associated with severe RSV disease have been described and include prematurity, chronic lung disease and congenital heart disease, weakened immune systems or neuromuscular disorders (2, 5). In addition, younger age, especially infants < 6 months are also at risk for severe RSV infection (5). Two to three out of every 100 infants infected with RSV may require hospitalization, however treatment remains supportive (2). At the time of this study, the only therapeutic option is ribavirin, although its use is controversial, and it is not recommended in many countries (6). At the time this study was initiated, RSV prophylaxis was mainly recommended for high-risk infants, to provide protection against RSV infection, in the form of monthly intramuscular injections throughout the RSV season. With the relatively recent approval of new options for RSV prophylaxis, several countries have updated their recommendations to include prophylaxis for all infants (7, 8).

In temperate climates RSV outbreaks typically occur in Autumn and Winter. In the Northern Hemisphere, epidemics usually occur from October to April, with a peak in January or February. In the Southern Hemisphere, epidemics occur from May to September, with a peak in June (9). On the other hand, RSV circulation occurs all year round in tropical regions.

The management of RSV presents numerous challenges for both HCPs and patients/caregivers, not least in terms of the variable seasonality according to the region, but also the lack of tools to predict which infants will develop severe disease requiring hospitalization or even pediatric intensive care unit management.

The lack of available treatment options may lead to reluctance from HCPs to test for the virus, and in some countries there is lack of readily available rapid testing (10, 11). Knowledge gaps amongst some primary care physicians regarding risk factors, epidemiology, and management of infants at risk of RSV may also contribute to challenges in infants receiving follow-up doses of RSV prophylaxis (12). Additionally, variations in populations eligible for RSV prophylaxis exist between national guidelines leading to inconsistencies in management across countries (13). Finally, increased workload during RSV season has led to some HCPs experiencing stress and exhaustion (11).

Many caregivers have limited awareness of RSV, and in the USA, 43% of caregivers of hospitalized infants with RSV infection, had not heard of the virus prior to their infant's illness (14). Additionally, adherence to follow up appointments may be reduced by caregiver misconceptions that RSV prophylaxis is a vaccine or due to geographic barriers to accessing clinics (15). Caregivers of hospitalized children with RSV may experience high levels of stress and anxiety and one study estimated that Health-related Quality of Life in infants infected with RSV, and their parents, reduced by 38% during the week after RSV diagnosis (10, 16). Concerns about the need for injections or potential side effects and needle burden may also be barriers to receiving prophylaxis (15). Finally, RSV presents a financial burden for caregivers, and it has been estimated that in the UK, the annual out-of-pocked cost of RSV illness in children under 5 years is £1.5 million (10, 17).

The objectives of this study were to:

1. Identify and prioritise international best practice approaches to RSV care in the pediatric and neonatal populations globally; and
2. Discuss methods to replicate these best practice interventions across other centers to improve patient outcomes.

## 2 Materials and methods

The study followed a four-step methodology:

### 2.1 Literature review

A comprehensive literature review was conducted to map the global RSV care pathway, identify challenges and unmet needs, and evidence-based best practice care for RSV across the pathway.

This involved:

• Academic literature search focusing on peer-reviewed resources. The search terms, all combined with “RSV” and “pediatric”, included: Clinical guidelines, Standard of care, Management, Prevention, Initiation, Continuation, Challenges in management, Unmet needs, Disease burden, In season/out of season management, Patient pathway, Identification.
• Grey literature search focusing on non-peer-reviewed articles and other supporting documents to supplement the academic literature review. The search terms, all combined with “selected countries in scope”, included: RSV pediatric clinical guidelines, RSV pediatric treatment pathway, RSV pediatric best practice, RSV pediatric management pathway, RSV pediatric management challenges, Patient support, Second season care.

Search tools such as PubMed were used to identify relevant publicly available academic literature, whilst Google and Google Scholar facilitated the search for grey literature and other publicly available sources. A range of documents including policy documents, reports, and articles were reviewed. Only English-language sources were considered and sources over 5 years old were excluded.

### 2.2 Center identification

Ten countries were selected for inclusion, and across these countries, a longlist of 30 centers was developed. Centers were identified based on pre-determined criteria to ensure a representative view of RSV care across the countries.

The criteria used were:

• Type of center e.g., public vs. private funding, size (i.e., number of beds), delivery setting for subsequent doses of RSV prophylaxis
• Focus on RSV research and trials
• Local or national contributions to the RSV guidelines/recommendations
• Key initiatives e.g., identification of second season infants
• Geographic spread e.g., urban vs. rural ecosystems

One center from each country was selected and invited to participate in the study. The departmental lead clinician from each center was contacted and invited to a briefing call outlining the study's purpose, data collection methods, and expected outputs. Center leads were then asked to confirm their participation.

See Table 1 in appendix for list of participating centers.

**Table 1:** Participating centers.

Country	Center
Austria	Medical University of Vienna, Comprehensive Center for Paediatrics
Brazil	Clinical Hospital of the Federal University of Rio Grande do Sul (UFRGS)
Chile	Complejo Asistencial Dr. Sotero del Rio
Colombia	Fundación Cardiovascular de Colombia
Italy	Institute for Maternal and Child Health IRCCS Burlo Garofolo
Japan	Japanese Red Cross Kyoto Daiichi Hospital
Malaysia	Hospital Canselor Tuanku Muhriz UKM
Poland	Katedra i Klinika Neonatologii
Turkey	Hacettepe University Faculty of Medicine
Saudi Arabia	Maternity and Children Hospital Al-Ahsa

### 2.3 Observation of best practice interventions

Best practice interventions in neonatal and pediatric RSV care delivery were observed through in-person or remote visits to participating centers. A semi-structured interview guide was developed to facilitate the collection of qualitative insights from HCPs across various disciplines, including neonatologists, pediatricians, pulmonologists, cardiologists, virologists, specialist nurses, and pharmacists. Interviews were conducted primarily in English, with translators utilised when necessary. Forty interviews were conducted, and each interview lasted approximately 45 min.

The following themes were covered:

• Introductions and overview of the study objectives
• Center and RSV unit specifics
• RSV care team roles
• Challenges in patient management
• Awareness and identification of at-risk infants
• Initiation of care
• Monitoring and follow-up care
• Caregiver education and empowerment
• Use of technology in RSV care delivery
• Wrap-up

Thematic analysis was conducted on interview insights from each country to identify best practice interventions. Triangulation within interviews ensured that the identified interventions were supported by multiple HCPs. These interventions were subsequently documented in ten center-specific reports. A comprehensive list of observed practices was compiled based on these reports.

### 2.4 Expert review and prioritization of best practice interventions

The longlist of identified best practice interventions was critically reviewed by a panel of 5 RSV experts, including HCPs and a patient group representative. Each individual intervention was discussed, and through consensus, it was assigned a high, medium, or low ranking against each of the two criteria. The combined ranking determined the intervention's overall priority level. The “prioritized” interventions were those that received a combined “high” ranking.

The expert group prioritized best practice interventions, based on:

• The degree to which the intervention impacts patient care and addresses current unmet needs in RSV prevention and treatment; and
• How feasible the intervention is to implement at other centers.

Ethical approval was not required for this study as no patient identifiable data was collected. Patients were not involved in this study.

## 3 Results

In total, 57 unique best practice interventions were observed and documented across the 10 centers and categorized into a best practice theme. Of these 57 unique best practice interventions, 16 of these were prioritized by the expert group.

See Table 2 in appendix for Longlist of unique best practice interventions and Table 3 for Prioritized best practice interventions.

**Table 2:** Longlist of best practice interventions observed.

Best practice theme	Best practice intervention
Caregiver education and engagement	In-person caregiver education
	Written education materials for caregivers
	Clinician/caregiver relationships
	Educating caregivers and the wider public
	Enforcing hygiene protocols for caregivers
	Raising public awareness
HCP education	Training the workforce
	Education for community HCPs
	Education of other centers
	Tailored training for midwives and junior nurses
	Educating primary care HCPs
	Visual reminders of RSV targeted at HCPs
	Tailored training for junior workforce
	RSV awareness amongst MDT
	Kyoto Paediatrician Committee
HCP-led RSV prophylaxis services	Outpatient follow-up clinic
	Ambulatory care center
	Reminders ahead of RSV prophylaxis appointments
	Dedicated outpatient RSV prophylaxis nurse
	Pulmonologist-led virtual clinic
	Home visits
	Home hospitalisation programme
	Home visits and transport support
Protocols and ways of working	Collaboration with cardiologist
	Collaborative MDT
	Distribution of epidemiology data
	Established relationship between neonatologist and virologist
	Strong focus on research
	Adaptation of national guidance for local needs
	Dedicated RSV nurse
	Pharmacist-led patient prioritisation process
	Specialist pharmacists on the NICU
	Collaborative emergency ward MDT
	Named preterm infant lead
	RSV prophylaxis guideline development
	Triaging infants on the emergency ward
	Adaptation of national guidance to local needs
	Isolation rooms
	Rapid screening test for RSV
	Monitoring RSV prophylaxis usage
	Triaging infants on the NICU
	Care coordinator
	Adherence to national guidelines
	Collaborative neonatology ward
	Seclusion of infectious patients
	Strong links to local network
	Defined care pathway between NICU and paediatric ward
	Reminders ahead of RSV prophylaxis appointments
Technology and innovation	Digitising patient records for continuity of care
	Neonatologist-led telemedicine
	Electronic healthcare records
	Paediatrician-led telemedicine services
	PROMESA programme
	Remote monitoring of clinical status
	Remote video consultations
	Digital RSV programme
	Digital database to track cardiology infants

**Table 3:** Prioritized best practice interventions.

Best practice theme	Best practice intervention
Caregiver education and engagement	In-person guidance for caregivers
	Written educational materials for caregivers
HCP education	Educating junior workforce on RSV
	Educating other centers
	Educating primary care HCPs
HCP-led RSV prophylaxis services	Instant messaging telephone services
	Outpatient prophylaxis clinic
	Reminders ahead of RSV prophylaxis appointments
	Home visits
Protocols and ways of working	Collaborative multidisciplinary care
	Dedicated RSV nurse
	Specialist pharmacists on the NICU
Technology and innovation	Electronic Healthcare Records (EHR)
	Database to track high-risk infants
	Remote video consultations
	Remote monitoring of clinical status

The 5 best practice themes are as follows:

### 3.1 Caregiver education and engagement

Timely caregiver education is important to raise awareness about the risks associated with RSV infection. This is particularly important when at-risk infants are born and can include general guidance to caregivers for RSV prevention, signs and symptoms of RSV, and required actions in case of suspected RSV infection. In addition, caregiver education is important to reduce misconceptions that RSV prophylaxis is a vaccine, which in turn, may help to improve adherence to prophylaxis appointments. Ultimately, providing caregivers with timely education equips them with the tools required to provide their infants with quality care outside of hospital settings.

Across the centers, 6 unique interventions were observed, 2 of which were prioritized by the expert group.

Prioritized best practice interventions aligned to this theme are described below.

#### 3.1.1 In-person guidance for caregivers

Members of the multidisciplinary team (MDT) provide in-person counselling to caregivers of at-risk infants. This can be during hospital admission and/or upon discharge and aims to:

• Address caregiver concerns or questions
• Educate caregivers about the use of RSV prophylaxis, empowering them to make informed decisions for their infants and minimise risk of RSV infection
• Reduce misconceptions about RSV prophylaxis
• Develop a trusted relationship between HCPs and caregivers

Guidance is particularly impactful if provided by the different members of the MDT whom caregivers encounter during their infant's hospital stay (e.g., neonatologists, pediatricians, nurses, pharmacists).

#### 3.1.2 Written educational materials for caregivers

Written educational materials (e.g., leaflets, brochures, or electronic resources) can be shared with caregivers at their infant's discharge. These materials include information on signs and symptoms of RSV and techniques for managing at-risk infants in the community (e.g., social factors, hygiene measures). Materials should also provide contact information for outpatient support services, details of community services available for caregivers, and signpost to parent support organizations and charities.

### 3.2 HCP education

Provision of continuous evidence-based education for all HCPs involved in the RSV pathway helps to provide high-quality care. Education can focus on RSV seasonality, identification of at-risk infants, and eligibility for prophylaxis. This education may extend to HCPs outside of the hospital setting, notably in primary care settings.

Across the centers, 9 unique interventions were observed, 3 of which were prioritized by the expert group.

Prioritized best practice interventions aligned to this theme are described below.

#### 3.2.1 Educating junior workforce on RSV

Education sessions for the junior workforce may be led by neonatologists, pediatricians, or specialized nurses to raise awareness about RSV seasonality, signs and symptoms of RSV and the disease course, RSV prophylaxis eligibility requirements, and management of suspected RSV infection.

Regular emails may also be sent to the entire workforce with updates regarding RSV guidelines and protocols, reminders about the upcoming RSV season, and to highlight additional resources available within the hospital.

#### 3.2.2 Educating other centers

Learnings can be shared with other centers, outside of the hospital setting, which provide RSV care. This may be in the form of a congress, scientific publication, or via virtual or in-person learning sessions and aims to:

• Discuss complex clinical cases
• Provide updates on regional epidemiology of RSV
• Reinforce knowledge on regional RSV protocols and guidelines
• Share insights from successfully implemented initiatives to improve patient identification or outcomes
• Strengthen the network of physicians and allied HCPs providing care to similar patient cohorts

#### 3.2.3 Educating primary care HCPs

Neonatologists or neonatal nurses provide teaching for family pediatricians and primary care HCPs about the importance of the use of RSV prophylaxis, and when to refer infants to the hospital for care. This may take the form of working groups, seminars, mailing lists or bulletins, and aims to:

• Enhance management of a-risk infants within the community setting
• Discuss complex clinical cases
• Provide updates on regional epidemiology of RSV
• Reinforce knowledge on regional RSV protocols and guidelines

### 3.3 HCP-led RSV prophylaxis services

Ensuring at-risk infants are protected ahead of the RSV season may require additional support services provided by the care team. Instant messaging services, or home visits, provide greater accessibility to the RSV care team and facilitates more personalized, and informed care for caregivers once their infant has been discharged.

Establishing outpatient RSV prophylaxis clinics allows for access to necessary follow-up RSV prophylaxis doses, helping to improve adherence to these appointments.

Across the centers, 8 unique interventions were observed, 4 of which were prioritized by the expert group.

Prioritized best practice interventions aligned to this theme are described below.

#### 3.3.1 Instant messaging telephone services

Access to third-party instant messaging platforms facilitates faster and more convenient communication between caregivers and HCPs. This allows caregivers to securely discuss their infant's care, receive timely guidance from HCPs, and schedule video follow-up consultations. However, any platforms must be compliant with local data protection laws and ensure patient confidentiality is maintained.

#### 3.3.2 Outpatient prophylaxis clinic

At-risk infants may receive monthly doses of RSV prophylaxis in outpatient clinics which may be led by specialists (e.g., neonatologists, pediatricians) or allied HCPs (e.g., pharmacists or nurses). During these appointments, nurses will administer RSV prophylaxis, provide education to caregivers, and address any caregiver concerns or questions. In some instances, these outpatient RSV prophylaxis clinics may run in tandem to general follow-up appointments.

#### 3.3.3 Reminders ahead of RSV prophylaxis appointments

Reminder notifications sent to caregivers ahead of their infant's follow-up appointment promote adherence to the appointment. Reminders may be sent via a phone call from nurse/administrative staff, an automated text alert, automated email, mobile app notification, or posted letter.

#### 3.3.4 Home visits

Home visits led by neonatologists, pediatricians, or nurses are conducted once at-risk infants have been discharged. Visits may incorporate a routine check-up such as blood pressure monitoring, measuring oxygen saturations, or counselling for caregivers on preventative hygiene measures. In some instances, home visits may also include administration of RSV prophylaxis led either by specialists or allied HCPs. Particularly in low-middle income countries, home-visits offer HCPs an opportunity to provide additional guidance to caregivers on RSV care.

### 3.4 Protocols and ways of working

Establishing evidence-based guidelines ensures best practices are followed within clinical practice. This promotes effective operation of the available facilities and ensures ongoing monitoring and evaluation of care delivery.

Across the centers, 25 unique interventions were observed, 3 of which were prioritized by the expert group.

Prioritized best practice interventions aligned to this theme are described below.

#### 3.4.1 Collaborative multidisciplinary care

Provision of diverse multidisciplinary care allows for more rapid intervention and tailored support. MDTs comprising neonatologists, pediatricians, nurses, and pharmacists meet regularly to discuss the best approach to patient care, prioritization of the most critical patients requiring specialist care, and agreeing individual patients' eligibility for RSV prophylaxis. Cardiologists and pulmonologists may also be involved in MDT discussions to advise on patients with congenital heart disease or bronchopulmonary dysplasia.

#### 3.4.2 Dedicated RSV nurse

A dedicated RSV nurse may be appointed to manage services associated with RSV care, including:

• Provision of specialized care within the Neonatal Intensive Care Unit (NICU)
• Oversight and management of the outpatient RSV prophylaxis clinic
• Delivery of caregiver education
• Promotion of RSV awareness initiatives in the hospital
• Provision of tailored training for the workforce

#### 3.4.3 Specialist pharmacists on the NICU

A dedicated pharmacist may be responsible for providing patient care in the NICU, including supporting patient discharge, through provision of guidance and identification of infants eligible for RSV prophylaxis. A dedicated pharmacist may provide support through:

• Provision of specialized care within the NICU
• Oversight and management of the outpatient RSV prophylaxis clinic
• Storage of RSV prophylaxis
• Provision of education on RSV prophylaxis
• Procurement of RSV prophylaxis in line with hospital formulary and budget
• Provision of tailored training for the workforce

### 3.5 Technology and innovation

Digital services are increasingly offered to address various challenges in care delivery and optimize patient experience (e.g., electronic healthcare records, mobile app monitoring services). These developments have been particularly impactful to reduce geographical barriers to accessing care (e.g., remote video consultations, remote monitoring of clinical status).

Across the centers, 9 unique interventions were observed, 4 of which were prioritized by the expert group.

Prioritized best practice interventions aligned to this theme are described below.

#### 3.5.1 Electronic healthcare records (EHR)

Deployment of an EHR across the hospital, which can be accessed by all members of the MDT, optimizes day-to-day workflow, and removes need for paper-based management of patients. Information stored within the EHR may include lab reports, imaging reports, past medical history including allergies, communications with caregivers, and RSV prophylaxis status.

#### 3.5.2 Database to track high-risk infants

A digital database which contains information about high-risk infants admitted with RSV allows nurses to follow up with caregivers on their infant's immunoprophylaxis status. Certain MDT members may be elected to collect data such as patient characteristics and the database is reviewed periodically (e.g., quarterly) by relevant team members to identify areas for improvement and conduct analysis.

#### 3.5.3 Remote video consultations

Select patients are followed up via video consultations, rather than face-to-face consultations within the center to reduce geographic barriers to accessing clinician support and advice. Remote video consultations can also be scheduled for non-routine check-up appointments (e.g., drop-in clinics for advice).

#### 3.5.4 Remote monitoring of clinical status

Access to a third-party mobile application facilitates faster and more convenient monitoring of high-risk infants, particularly for patients residing in rural locations. Transition of care to different settings (i.e., if follow-on doses are administered in a different center) is also facilitated if medical records can be accessed via the app. Caregivers can submit measurements such as weight, feeding routine, oxygen saturation for regular review by clinicians. However, any app must be compliant with local data protection laws and ensure patient confidentiality.

## 4 Discussion

The study identified 16 unique best practice interventions across 5 best practice themes following literature review, center visits and an expert group review.

The identified best practice interventions can be replicated by centers globally to address challenges specific to their care setting and improve the quality of care delivered to their neonatal and pediatric populations. To identify which best practices to implement, it is recommended that HCPs assess their center by considering the following questions: Could more be done to increase participation in awareness initiatives amongst the wider care community and general public? Would it be beneficial to offer a wide range of dedicated support programs for parents and caregivers? Could more be done to monitor adherence and follow up management? Could more be done to review ways of working for the identification and management of patients? Would it be beneficial to offer a wide range of outreach services?

When planning to implement the selected best practice interventions it will be key for care settings to consider the relative cost, effort level and impact of each intervention. For each intervention, HCPs should consider:

• Target audience (e.g., caregivers, junior doctors, allied HCPs, at-risk infants)
• Timeframe (e.g., monthly meetings)
• Staffing requirements (e.g., HCPs, administrative staff)
• Training requirements (e.g., training for HCPs on new software, home visit protocols)
• Resources requirements (e.g., access to private consultation rooms)
• Potential costs (e.g., costs to print materials)
• Key Performance Indicators (KPIs) to monitor and track success (e.g., engagement levels, satisfaction scores)
• Potential impact (e.g., enhanced awareness of RSV amongst caregivers, improved adherence to follow-up doses of RSV prophylaxis)

It will be important to note that the role of HCPs and allied HCPs can vary across different countries which may limit the replicability of certain interventions or may require the interventions to be adapted accordingly.

A document has been developed to support centers in replicating the interventions in their own settings. The next steps for the project group are to make this document available for use by HCPs.

It was recognized that there were limitations to the study methodology. Firstly, caregivers were not interviewed during this study, limiting the patient and caregiver perspective on challenges captured in the RSV care pathway. We recommend that future studies incorporate caregiver input to better understand their needs, perceptions, and challenges. Secondly, one center was interviewed per country therefore interview insight may not be representative of the wider healthcare system in that country. The centers selected were tertiary care centers, which may have introduced selection bias favoring better-equipped hospitals. This could potentially limit the generalization of the interventions to lower-complexity or resource-constrained healthcare settings. To try and reduce this bias, the study included a mix of centers from both urban and rural areas.

Globally, there are continual efforts to advance RSV care. Since the initiation of this study, the UK government announced a new NHS vaccination programme against RSV (18). The programme aims to protect infants by offering a single dose of an RSV vaccine to all women who are at least 28 weeks pregnant. Similarly, maternal RSV vaccines have been approved for use in Japan, USA and other countries (19, 20).

Additionally, the study has been extended to an additional two countries with lower-complexity or resource constraints. At the time of writing, these center visits were commencing.

## 5 Conclusion

This global study identified and evaluated interventions that may improve patient outcomes and quality of care for RSV disease in the pediatric neonatal population. The next steps will be to disseminate and implement these examples of best practices in healthcare systems and settings globally.

## Data Availability

The original contributions presented in the study are included in the article/Supplementary Material, further inquiries can be directed to the corresponding author.
